# Mitochondrial protein Fus1/Tusc2 in premature aging and age-related pathologies: critical roles of calcium and energy homeostasis

**DOI:** 10.18632/aging.101213

**Published:** 2017-03-26

**Authors:** Roman Uzhachenko, Kelli Boyd, Danyvid Olivares-Villagomez, Yueming Zhu, J. Shawn Goodwin, Tanu Rana, Anil Shanker, Winston J.T. Tan, Tanya Bondar, Ruslan Medzhitov, Alla V. Ivanova

**Affiliations:** ^1^ Department of Biochemistry and Cancer Biology, School of Medicine, Meharry Medical College, Nashville, TN 37208, USA; ^2^ Department of Pathology, Microbiology and Immunology, Vanderbilt University School of Medicine, Nashville, TN 37232, USA; ^3^ Department of Radiation Oncology, Feinberg School of Medicine, Northwestern University, Chicago, IL 60611, USA; ^4^ Host-Tumor Interactions Research Program, Vanderbilt-Ingram Cancer Center, and the Center for Immunobiology, Vanderbilt University, Nashville, TN 37235, USA; ^5^ Department of Surgery, Section of Otolaryngology, Yale University School of Medicine, New Haven, CT 06510, USA; ^6^ Department of Immunobiology, Yale University School of Medicine, New Haven, CT 06510, USA; ^7^ Present address: Division of Clinical Pharmacology, Vanderbilt University Medical Center, Nashville, TN 37235, USA

**Keywords:** Fus1/Tusc2, mitochondrial Ca^2+^, aging and age-related diseases, chronic inflammation, calcium response, mitochondrial respiration

## Abstract

Decreased energy production and increased oxidative stress are considered to be major contributors to aging and aging-associated pathologies. The role of mitochondrial calcium homeostasis has also been highlighted as an important factor affecting different pathological conditions. Here, we present evidence that loss of a small mitochondrial protein Fus1 that maintains mitochondrial homeostasis results in premature aging, aging-associated pathologies, and decreased survival. We showed that Fus1KO mice develop multiple early aging signs including lordokyphosis, lack of vigor, inability to accumulate fat, reduced ability to tolerate stress, and premature death. Other prominent pathological changes included low sperm counts, compromised ability of adult stem cells to repopulate tissues, and chronic inflammation. At the molecular level, we demonstrated that mitochondria of Fus1 KO cells have low reserve respiratory capacity (the ability to produce extra energy during sudden energy demanding situations), and show significantly altered dynamics of cellular calcium response.

Our recent studies on early hearing and memory loss in Fus1 KO mice combined with the new data presented here suggest that calcium and energy homeostasis controlled by Fus1 may be at the core of its aging-regulating activities. Thus, Fus1 protein and Fus1-dependent pathways and processes may represent new tools and targets for anti-aging strategies.

## INTRODUCTION

Ample evidence suggests that defects in mitochondrial activities occur during aging [1-4]. However, many aspects of the role of mitochondria in aging remain poorly understood. A critical link between oxidative stress, aging, age-related diseases and lifespan has been supported by several mouse models that target or overexpress antioxidant (AO) proteins [5-10] or regulators of AO pathways [11-13]. Remarkably, other mouse models demonstrate that alleviation of oxidative stress is not always linked with the extension of lifespan [14-16]. Moreover, it was shown that the outcome largely depends on targeting AO proteins to certain cellular compartments. Thus, mice with transgenic catalase targeted to the peroxisome (PCAT), nucleus (NCAT), or mitochondrion (MCAT) showed drastically different lifespans [17, 18]. The largest effect on lifespan was found in MCAT animals (20% increase) followed by a more modest effect in PCAT animals, and no significant change in NCAT animals [17, 18]. Therefore, balancing mitochondrial reactive oxygen species (ROS) production with other mitochondrial activities should be at the crux of anti-aging and lifespan extension strategies.

Although mitochondria are vital for energy production, increasing evidence shows that they also regulate a wide range of cellular signaling pathways and homeostasis via a process called retrograde signaling mediated by molecules produced in mitochondria, i.e. ATP, ROS, Ca^2+^, NO, NAD, etc. [19-21]. To understand the role of mitochondrial homeostasis and signaling in aging and age-related diseases and to develop anti-aging strategies, novel mouse models of premature aging driven by different types of mitochondrial dysfunction will be instrumental.

We previously established Fus1/Tusc2 as a tumor suppressor, immune modulator and regulator of oxidative stress [22-26]; however, only recently have we started understanding the molecular mechanisms of these activities. Fus1 is a small (110 aa) nuclear-encoded mitochondrial protein. It has no transmembrane domains and is predicted to be a globular protein situated in the mitochondrial matrix. Our *in silico* and *in vitro* studies suggest that Fus1 belongs to a group of Ca^2+^/myristoyl switch-like proteins and regulates mitochondrial Ca^2+^ homeostasis and, thus, Ca^2+^-coupled processes in cells. We demonstrated that Fus1 loss leads to inefficient accumulation of Ca^2+^ in mitochondria, which profoundly alters ROS production, mitochondrial membrane potential (MMP), and mitochondrial dynamics [25, 27]. At the organismal level, even in young Fus1 KO mice we identified multiple alterations linked to aging-associated processes such as increased ROS production and decreased AO defense [26], insufficient DNA damage response [28], and perturbed inflammatory response to noxious stimuli [23, 25, 27].

Here, we present numerous evidence of premature aging, shorter survival, chronic inflammation, altered capability of Fus1 KO cells to repopulate tissues, and earlier development of aging-related pathologies in Fus1 KO mice. At the molecular level, we present the details of mitochondrial dysfunction in Fus1 KO cells, such as altered Ca^2+^ response and low stress-induced mitochondrial respiration (respiratory reserve capacity) that, in combination with oxidative stress [25, 29-32], may precipitate early aging, aging-associated pathologies and death. We also present *in silico* data on strong association of Fus1 expression with a subset of genes involved in age-related diseases. Interestingly, *in silico* data on Fus1 expression in aging human tissues showed significant age-dependent decrease of Fus1 expression in muscle tissues that coupled with our data suggest that Fus1 may have clinical significance as an anti-aging molecule.

## RESULTS

### Fus1 KO mice die earlier

**Figure 1:**
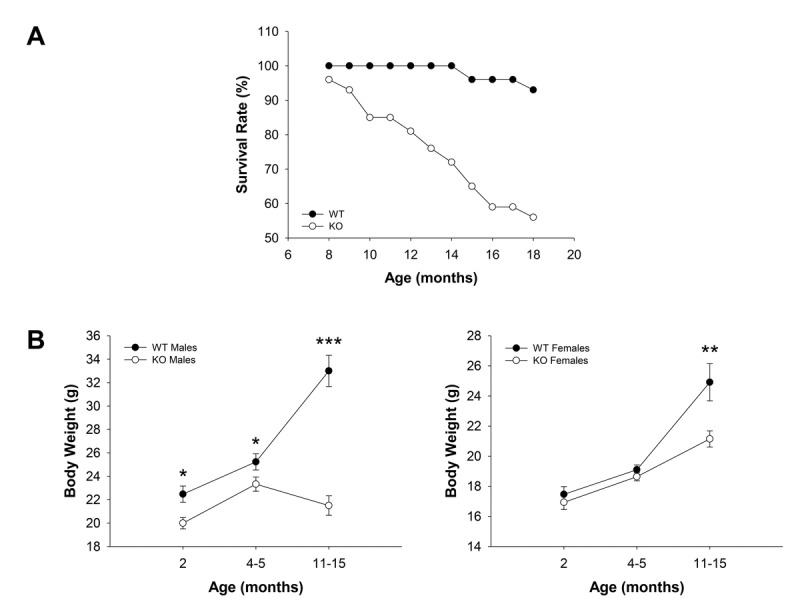
Survival and age-dependent dynamics of body weights in WT and Fus1 KO mice (**A**) Survival curves for WT (*n* = 30) and Fus1 KO (*n* = 54) mice from 8 to 18 months of age. (**B**) Age-dependent changes in body weights of WT and Fus1 KO mice. *p-value ≤ 0.05; **p-value ≤ 0.005; ***p-value ≤ 0.0005 (Student's *t*-test, 2-sided unpaired). Number of mice used in the analysis: 2 m.o. males, *n* = 11-12/group; 2 m.o. females, *n* = 15-18/group; 4-5 m.o. males, *n* = 17-18/group; 4-5 m.o. females, *n* = 8/group; 11- 15 m.o. males, *n* = 10/group; 11- 15 m.o. females, *n* = 9-13/group. Data expressed as mean ± SEM.

WT (*n* = 30) and Fus1 KO (*n* = 54) mice of both genders maintained in the same animal room were observed between 8 and 18 months old (m.o.) and spontaneous death among groups was recorded. While only 2 mice (7%) of 15 and 18 months of age within the WT cohort died during the observation period, 24 mice in the KO cohort (44%) died with their age of death varying between 8 and 18 months old (Fig. 1A). No gender-related difference in time of death was noticed. Biopsy carried out on the mice which tissues were suitable for histopathological examination showed no obvious overt signs of disease that could be interpreted as life-threatening except for two Fus1 KO mice (12 and 14 months old) that developed acute autoimmune syndrome including polyarteritis, acute renal tubular necrosis and acute glomerular thrombosis that could be the cause of death. This pathology in Fus1 KO mice was expected due to their susceptibility to development of autoimmune symptoms at middle age [23]. However, in most cases the cause of death was not determined.

### Inability to accumulate fat with age in Fus1 KO mice

In addition to storing energy, fat is important in immune and endocrine function, thermoregulation, mechanical protection, and tissue regeneration [33]. Accumulation of fat is a characteristic feature of the growing body with maximal increase in fat tissue mass through middle age, which declines in old age [34]. Since changes in body weight reflect alterations in fat accumulation, we monitored age-dependent body weight changes in the population of WT and Fus1 KO mice. We found a statistically significant decrease in the body weight of Fus1 KO males in all age groups (2 m.o., 4-5 m.o., and 11-15 m.o.) as compared to WT males (Fig. 1B). Interestingly, although Fus1 KO males gained some weight between 2 and 4-5 months of age, further on they showed a persistent decline in body weight, suggesting that they reached a peak of their mature stage much earlier than WT mice, which still gained weight in the group of 11-15 m.o. males. In females, we observed a trend towards a decreased body weight in young (2 m.o.) and adult (4-5 m.o.) groups and a statistically significant weight decrease in 11-15 m.o. Fus1 KO as compared to age-matched WT females (Fig. 1B). However, unlike Fus1 KO males, females still continued to accumulate fat even at 11-15 months of age, although at a slower rate than WT females.

### Premature signs of systemic aging in Fus1 KO mice

**Figure 2:**
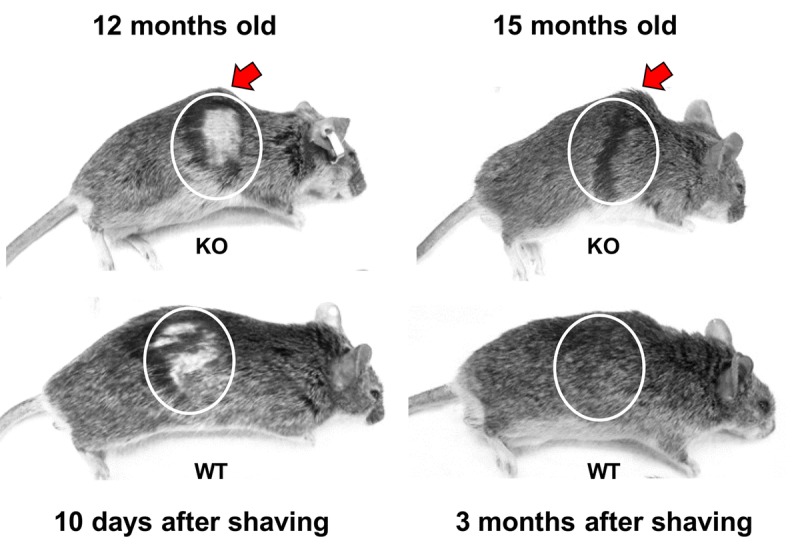
Premature signs of systemic aging in Fus1 KO mice KO mice (upper row) show signs of lordokyphosis (hunchbacked spine, pointed with arrow) and thinning of subcutaneous fat earlier than WT mice (bottom row). Also, hair-growth assay showed that 12 m.o. WT mice (*n* = 5) partially re-grew their hair at 10 days after shaving (bottom row) and had completely restored hair at 1 month after shaving while none of the KO mice (*n* = 5) showed hair growth at 10 days and all of them failed to close the shaved area even after 3 months. Shaving areas are circled.

We examined the possibility that the shortened lifespan of Fus1 KO mice could be a result of premature aging. Up to 6 months of age, Fus1 KO mice appear morphologically similar to WT mice. However, by 9-12 months, 50% of the Fus1 KO mice and no WT mice exhibited signs of lordokyphosis (hunchbacked spine) and an absence of vigor, which are characteristic signs of senescence (Fig. 2. and supplemental video). By 18 months, about 80% of Fus1 KO mice developed lordokyphosis.

Hair re-growth declines linearly as a function of age [35, 36]. Using a common hair-growth assay, we compared the ability of 12 m.o. WT and KO mice to re-grow hair. While all of the 12 m.o. WT mice (*n* = 5) partially re-grew their hair at 10 days post-shaving (Fig. 2) and had completely restored hair at 30 days post-shaving, none of the KO mice (*n* = 5) showed hair growth at 10 days and all of them failed to close the shaved area even at 3 months post-shaving (Fig. 2).

A reduced ability to tolerate stress is a hallmark of aging [37, 38]. Our observation on increased stress-sensitivity in young Fus1 KO mice aggravated by age were in-line with the premature aging phenotype of Fus1 KO mice. Thus, we found that Fus1 KO mice were more sensitive to the anesthetics avertin, pentobarbital and chloral hydrate than WT mice; hence, a reduced dose had to be used for Fus1 KO mice. Moreover, we observed that Fus1 KO mice of pup-bearing age were extremely sensitive to stress-inducing environments such as increased traffic and prolonged light exposure in the mouse room, which happened after installation of a new common hood in close vicinity to the WT and Fus1 KO mice mating cages. While frequency of pregnancies and maternal behavior of WT mice were not changed at these poor environmental conditions, frequency of pregnancies in Fus1 KO colony was drastically decreased. Moreover, all pups from occasional Fus1 KO litters born during this time died due to negligence of the mothers who did not feed the newborns. Interestingly, Fus1 KO mice resumed their normal breeding and maternal behavior shortly after their cages were put in the quiet and darker corner of the same room and supplied additionally with shredded paper and igloos for nesting.

### Low sperm count and reduced sperm motility in 10-12 m.o. Fus1 KO mice

**Figure 3:**
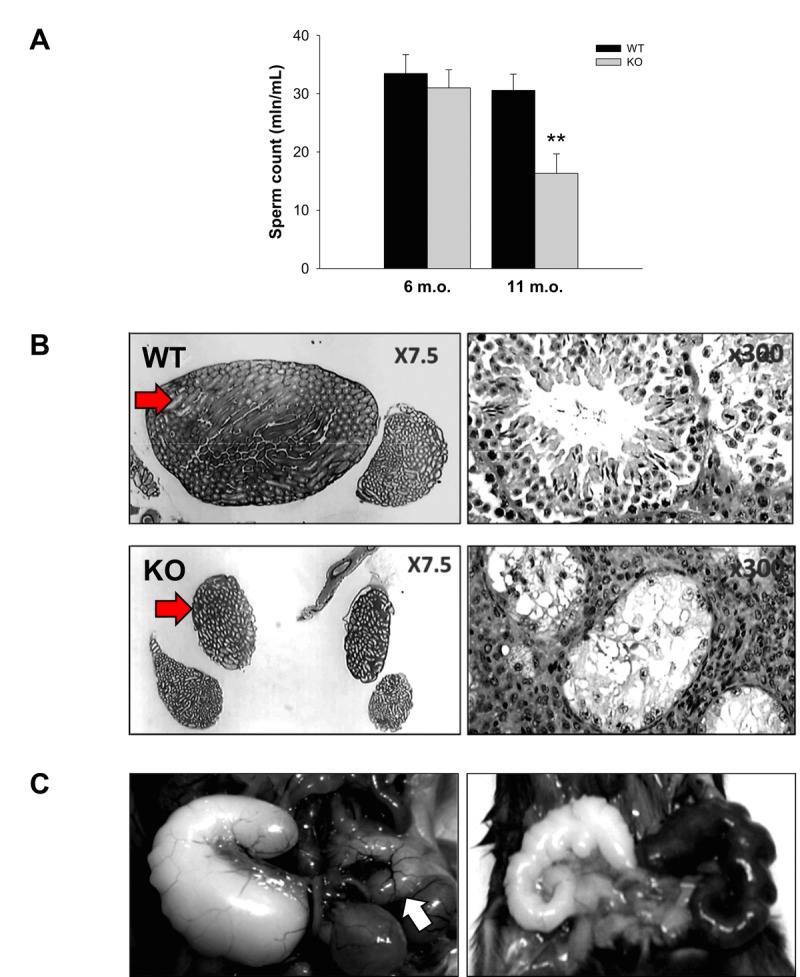
Prematurely developed low sperm count, occasional testes degeneration or vesicle enlargement observed in Fus1 KO but not in WT mice (**A**) Sperm count in WT and Fus1 KO mice of different ages (6 m.o.: WT mice, *n* = 10, KO mice, *n* = 9; 11-12 m.o.: WT mice, *n* = 6; KO mice, *n* = 9) revealed a premature sperm count decrease in 11-12 m.o. Fus1 KO mice; (**B**) Unilateral (not shown) and bilateral testes degeneration (shown at x7.5 and x300 magnification) were observed occasionally in adult Fus1 KO but not in WT mice. Arrows point to a normal testis from a WT mouse and a degenerated one from a Fus1 KO mouse; (**C**) Enlargement of seminal vesicles, an aging lesion that occurs spontaneously in some mice aged 24 mo or older were found in Fus1 KO mice of 16-20 months old. Lesions of different types were observed: asymmetrical vesicle enlargement (left), a normal size vesicle is shown by the arrow; symmetrical enlargement and discoloration of vesicles (right); symmetrical vesicle enlargement (not shown).

Decreased sperm concentration and motility are common signs of aging [39]. The sperm number in the cauda epididymis and the sperm velocity in aging WT and Fus1 KO males were analyzed using CEROS II Animal sperm analyzer (Hamilton-Thorne Research). While there was no difference between 6 m.o. WT and Fus1 KO mice (*n* = 6-8/group), a significant decrease in the sperm count and reduced sperm motility was observed in 11-12 m.o. Fus1 KO as compared to WT mice (Fig. 3A). Histopathological analysis of testes and caudas from KO mice showed that ~10% of aging animals had a symmetrical (Fig. 3B) or unilateral decrease (data not shown) in the size of the caudas and testis, which was not observed in age-matched WT males; some mice showed pronounced mineralization in these organs (data not shown).

### Enlargement of seminal vesicles in old Fus1 KO mice

Enlargement of seminal vesicles in male mice infrequently occurs spontaneously in senescent mice at 24 months of age or later [40]. We found that some KO males but not WT mice developed these aging lesions earlier in life (16-18 m.o.). We observed different types of lesions: asymmetrical vesicles enlargement (Fig. 3C), symmetrical enlargement (data not shown), and enlargement with discoloration (Fig. 3C).

### Altered capability of Fus1 KO adult stem cells to repopulate tissues

**Figure 4:**
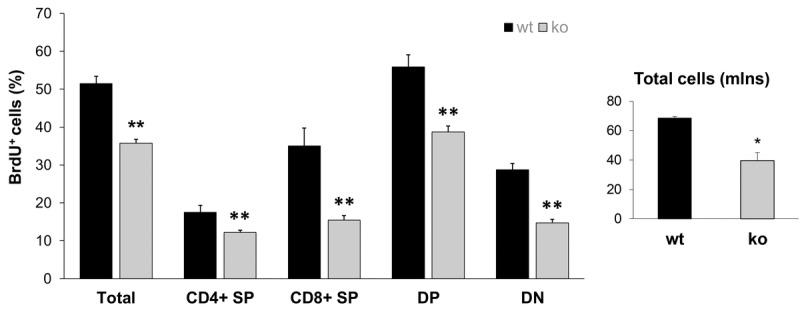
Lower proliferative capacity of Fus1 KO thymocytes revealed their lower renewal potential Proliferation capacity was estimated by calculating the ratio of BrdU-positive T cells to the total number of T cells. *p-value ≤ 0.05; **p-value ≤ 0.005 (Student's *t*-test, 2-sided unpaired). Data expressed as mean ± SEM (*n* = 4 mice/group).

The most recent *Stem Cell Theory of Aging* [41, 42] considers the decreased ability of stem cells to replenish damaged tissue as the main cause of aging. Our recent study demonstrated that Fus1 KO tissues with high cellular turnover (GI crypt epithelial cells and melanocyte stem cells) have a compromised self-renewal ability after exposure to ionizing radiation [28]. The results of the hair re-growth test on aging animals presented in Fig. 2 are also in-line with the compromised self-renewal of Fus1 KO stem cells in hair follicles. Here, we compared the capacity of thymocytes to proliferate as a measurement of their renewal potential. Overall thymic size and cellularity is maintained by replenishing with bone marrow-derived progenitor populations [43]. Once in the thymus, thymocytes undergo differentiation and proliferation. We compared the overall T cell number with the number of proliferating (BrdU-positive) cells in the thymi of young KO and WT mice (*n* = 4/group). The total thymocyte numbers were significantly lower in KO mice in comparison to their WT counterparts. Moreover, when we analyzed the proliferation potential of different thymocyte populations, we observed lower BrdU incorporation in CD4^-^CD8^-^ (double negative), CD4^+^CD8^+^ (double positive), CD4^+^CD8^-^ (CD4 single positive) and CD4^-^CD8^+^ (CD8 single positive). These results suggest that the repopulating ability of KO T cell progenitors is altered (Fig. 4).

### 6 m.o. Fus1 KO mice have signs of chronic inflammation

**Figure 5:**
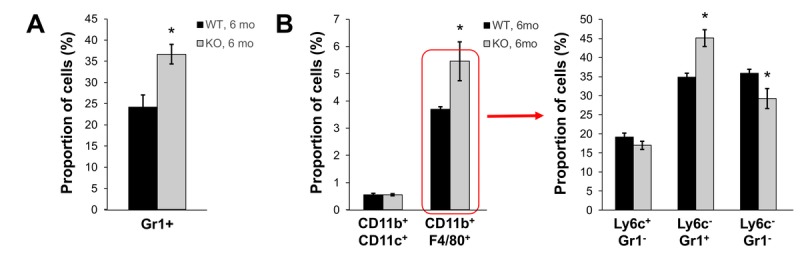
Fus1 KO mice (6 m.o.) show an increased number of inflammatory monocytes in peripheral blood and spleen *p-value ≤ 0.05; **p-value ≤ 0.005 (Student's *t*-test, 2-sided unpaired). Data expressed as mean ± SEM (*n* = 6 mice/group).

In our previous studies, we showed that young Fus1 KO mice have altered/exaggerated immune response to environmental and infectious stimuli [22, 25]. Moreover, middle-aged Fus1 KO mice, but not WT mice, were prone to developing an autoimmune SLE-like syndrome [23, 44]. We tested the hypothesis that Fus1 KO mice have low-grade chronic inflammation that helps them to clear non-lethal infections faster than WT mice at a young age [22], but plays a detrimental role and is involved in organismal aging when mice get older. We performed the analysis of peripheral and splenic myeloid and lymphocyte subsets in 6 m.o. WT and KO mice since this is the earliest age of the phenotypical manifestation of aging in Fus1 KO mice. In the lymphocyte compartments we did not find a prominent difference between WT and KO mice except for a significant decrease in CD3^+^ CD4^+^ T cells in Fus1 KO mice that we have already seen in young Fus1 KO mice, thus we did not consider it as an aging-associated change (data not shown). However, we found a significantly elevated number of Gr1^+^ myeloid cells in peripheral blood of Fus1 KO mice as compared to WT mice (37% vs 24% in KO *vs* WT, p ≤ 0.01, Fig. 5A). In the spleen, we detected a selective increase in the proportion of CD11b^+^F4/80^+^ macrophages (5.5 % vs 3.7% in KO vs WT, p≤ 0.05 Fig. 5B) while a fraction of CD11b^+^CD11c^+^ dendritic cells was similar in both WT and Fus1 KO mice (Fig. 5). After co-staining of CD11b^+^F4/80^+^ macrophages with additional markers Ly6c and Gr1, we found an almost 30% elevation of CD11b+F4/80+Gr1+ cells (p≤0.05), which corresponds to inflammatory monocytes but may also include phenotypic MDSC (myeloid-derived suppressor cells) (Fig. 5B) often associated with chronic inflammation or cancer [45].

### Defective mitochondrial respiration in Fus1 KO cells

The two basic theories of aging, the Free Radical Theory of Aging (FRTA) [46] and its later modification, the Mitochondrial Free Radical Theory of Aging [47, 48], agree that cumulative self-inflicted oxidative damage by ROS to mitochondrial DNA, proteins and lipids is one of the major causes of aging.

**Figure 6:**
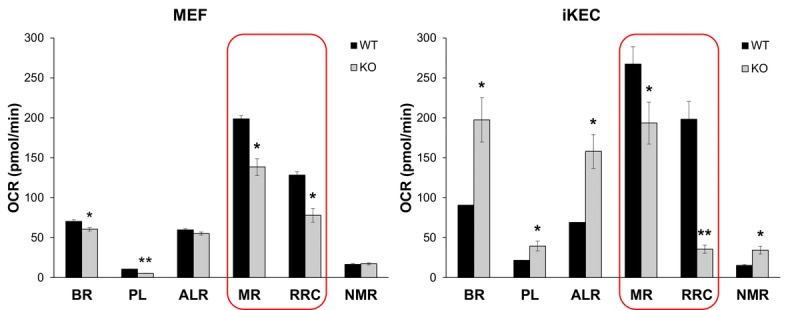
Fus1 loss results in a decreased maximal respiration and respiratory reserve capacity in primary MEFs and immortalized epithelial Fus1 KO cells High resolution respirometry oxygraph-2k (Oroboros Instruments) was used for analysis of cellular respiration. Abbreviations are as follows: OCR - oxygen consumption rate, BR - basal respiration rate, PL - proton leak rate, ALR - ATP-linked respiration; MR - maximal mitochondrial respiration, RRC – respiratory reserve capacity, NMR - non-mitochondrial respiration. Experiments were performed in triplicates. *p-value ≤ 0.05; **p-value ≤ 0.005 (Student's t-test, 2-sided unpaired). Data expressed as mean ± SEM.

Decline in mitochondrial activity, in turn, leads to enhanced ROS production that aggravates oxidative stress and mtDNA damage. We showed in several studies that Fus1 KO immune and epithelial cells produce higher levels of ROS at both basal and activated states [22, 25, 27]. Since unbalanced ROS production may be a result of defective mitochondrial respiration, we measured basal and stress-induced mitochondrial respiration in WT and Fus1 KO primary mouse embryonic fibroblasts (MEFs) and immortalized via serial passaging kidney epithelial cells (iKEC) using high resolution respirometry oxygraph-2k (Oroboros Instruments). We analyzed basal Oxygen Consumption rate (OCR) and stress-induced OCR after inhibition of different respiratory complexes. While primary WT and KO MEFs had similar rates of ATP turnover (ALR) and non-mitochondrial oxygen consumption (NMR), KO MEFs demonstrated significantly lower rates of basal respiration (BR) and proton leak (PL) (Fig. 6, left panel). Interestingly, iKEC had significantly higher rates of all these parameters (BR, PL, ALR and NMR) than the WT cells (Fig. 6, right panel) indicating that immortalized Fus1 KO epithelial cells have higher rates of mitochondrial respiration at steady state conditions. This difference in the rate of respiration between primary MEFs and immortalized KEC cells may be explained either by cell specificity, or by immortalization state of iKEC, in which an increased respiration may be a pre-requisite for immortalization of Fus1 KO cells. However, both Fus1 KO cell models showed a common mitochondrial deficiency, namely, a significantly lower maximal respiration (MR) and reserve respiratory capacity (RRC) as compared to WT cells (Fig. 6, left and right panels, outlined bars). MR and RRC levels reflect the ability of cells to produce additional ATP in response to increased energy demands. The fact that MR and RRC deficiency of Fus1 KO cells were not dependent on a cell type or immortalization status suggests an intrinsic defect in the bioenergetic reserve capacity in all Fus1 KO cells, which may be a cause of premature aging in Fus1 KO mice.

### Fus1 KO epithelial and fibroblast cells have perturbed Ca^2+^ dynamics

**Figure 7:**
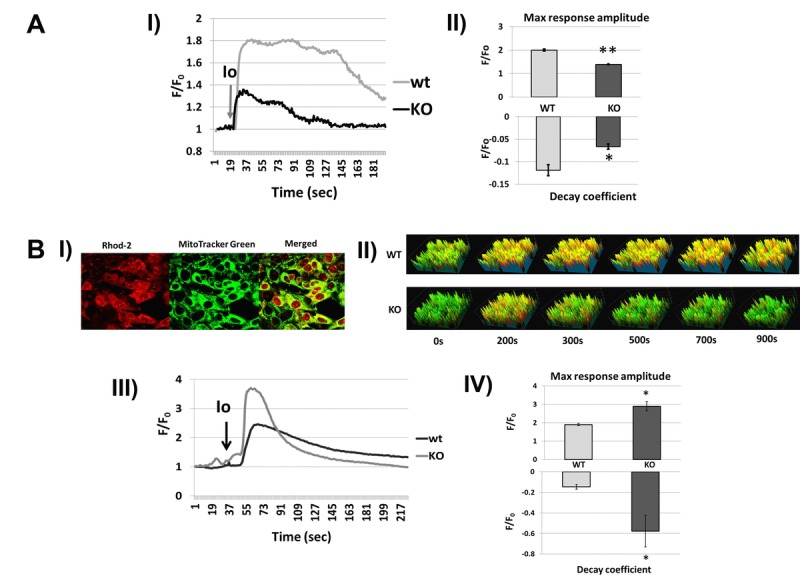

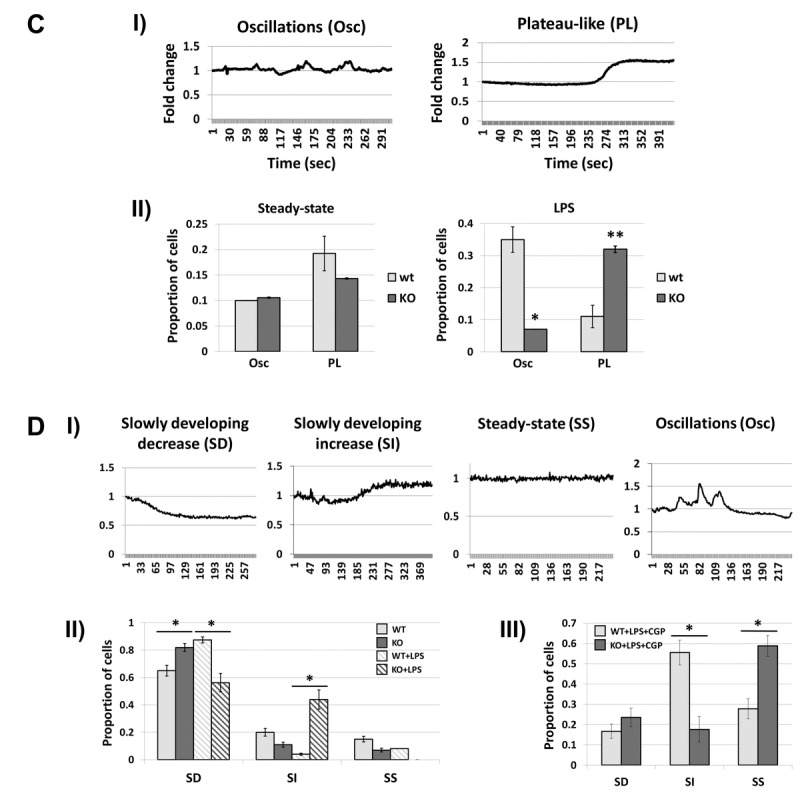
Fus1 KO cells show aberrant [Ca^2+^]c and [Ca^2+^]m responses to different stimuli that were improved by inhibiting the mitochondrial sodium/calcium exchanger (mNCX) (**A**) [Ca^2+^]c changes in Fus1 KO and WT iKEC in response to the calcium agonist Ionomycin. *Panel I* shows dynamic [Ca^2+^]c levels in WT and Fus1 KO iKEC after treatment with Ionomycin (Io, grey arrow) detected by Fura-2 Ca^2+^-sensitive fluorescent probe. Ratio 340/380 for Fura-2 in Io-stimulated iKEC (F) was normalized to the fluorescence value of control levels (without Io, Fo); *Panel II* shows parameters of [Ca^2+^]c response induced by Ionomycin in WT and Fus1 KO iKEC: maximal amplitude of response (upper section) and coefficient of decay phase (lower section). The level of statistical significance is designated as *p < 0.05, **p < 0.01. (**B**) changes in Fus1 KO and WT iKEC in response to Ionomycin. *Panel I* shows compartmentalization of Ca^2+^-sensitive fluorescent dye Rhod-2 (red) in iKE cells stained with mitochondria-specific dye MTG. Yellow color in the merged image represents staining of Rhod-2 in mitochondria. *Panel II* demonstrates snapshots showing Ionomycin-induced temporal changes of [Ca^2+^]m levels in WT and Fus1 KO iKEC double-stained with MTG/Rhod-2; *Panel III* shows [Ca^2+^]m dynamics profiles for WT and Fus1 KO iKEC obtained by double staining of iKEC with MTG/Rhod-2. Curves represent ratio of Rhod-2 fluorescence normalized to MTG fluorescence; *Panel IV* shows parameters of [Ca^2+^]m response induced by Ionomycin in WT and Fus1 KO iKEC: maximal amplitude of [Ca^2+^]m response (upper panel) and coefficient of [Ca^2+^]m decay phase after Ionomycin induction (lower panel). (**C**) Steady state and LPS-induced [Ca^2+^]c profiles in WT and Fus1 KO MEFs. *Panel I* demonstrates major patterns of [Ca^2+^]c responses in WT and Fus1 KO primary MEFs at steady state and after LPS treatment detected by Fura-2 Ca^2+^-sensitive fluorescent dye. *Panel II* demonstrates the proportion of cells with Osc- and PL-type [Ca^2+^]c responses at steady state and after treatment with LPS (100 ng/mL). (**D**) Dynamics of basal, LPS- and CGP-induced [Ca^2+^]m responses in WT and KO primary MEFs. *Panel I* shows major patterns of [Ca^2+^]m responses at steady state and after LPS treatment (100 ng/mL) detected by MTG/Rhod-2 co-staining. *Panel II* shows the proportion of cells with SD-, SI-, and SS-patterns of [Ca^2+^]m responses in WT and Fus1 KO MEFs at steady state and after treatment with LPS (100 ng/mL). *Panel III* demonstrates the proportion of cells with SD-, SI-, and SS-patterns of [Ca^2+^]m responses after co-treatment of MEFs with LPS (100 ng/mL) and CGP37157 (CGP), an inhibitor of mitochondrial of Na^+^/Ca^2+^ exchanger. *p-value ≤ 0.05; **p-value ≤ 0.005 (Student's *t*-test, 2-sided unpaired). Data expressed as mean ± SEM.

Earlier, we showed that Fus1 is involved in the regulation of Ca^2+^-mediated signaling in immune cells [31]. Considering the importance of Ca^2+^ homeostasis in aging [49, 56] we studied Fus1-dependent changes in Ca^2+^ dynamics in iKEC (immortalized kidney epithelial cells) and primary MEFs on a single-cell level using a wide-field and confocal microscopy approach. We evoked changes in Ca^2+^ by applying the Ca^2+^ agonist Ionomycin to iKEC and lipopolysaccharide (LPS) to MEFs. LPS, isolated from Gram-negative bacteria, normally induces a pro-inflammatory response in fibro-blasts preceded by elevation in cytosolic Ca^2+^ ([Ca^2+^]c) [57]. Fura-2, ratiometric Ca^2+^ indicator, was used to detect alterations in [Ca^2+^]c while mitochondrial Ca^2+^ ([Ca^2+^]m) dynamics was analyzed by co-staining of cells with MitoTracker Green (MTG; mitochondria-specific dye) and Rhod-2 (Fig. 7B, Panel I).

Fus1 KO iKEC treated with Ionomycin demonstrated a 45% lower amplitude of [Ca^2+^]c response and a 50% higher amplitude of initial [Ca^2+^]m elevation as compared to WT cells (Fig. 7A, Panel I). Another parameter, a coefficient of decay for [Ca^2+^]c response, which indicates a recovery of [Ca^2+^]c level after stimulus-induced rise, was also significantly different for both compartments, the cytosol and mitochondria (Fig. 7A, Panel II and 7B, Panel IV). The most pronounced difference was observed for [Ca^2+^]m decay: recovery to a steady-steady Ca^2+^ level after stimulation in Fus1 KO mitochondria occurred ~ 3 fold faster than in WT cells (Fig. 7B, Panel IV). Thus, Fus1 loss in iKEC leads to impairment of Ca^2+^ dynamics in both cytosolic and mitochondrial compartments.

Next, we studied Ca^2+^ changes in MEFs during early inflammatory response, which normally precedes expression of pro-inflammatory cytokines. First, we established the baseline Ca^2+^ fluctuations. Two major patterns of [Ca^2+^]c changes, plateau-like (PL) elevations and low-amplitude oscillations (Osc), were observed (Fig. 7C, Panel I). We compared a proportion of cells with these Ca^2+^ patterns in WT and KO MEFs before and after LPS stimulation. Although steady-state WT and KO MEFs evoked similar basal fluctuations of [Ca^2+^]c, LPS treatment resulted in a significant increase in cells with Osc pattern in WT but not in KO MEFs, which had a higher proportion of cells with PL response (Fig. 7C, Panel II). Analysis of [Ca^2+^]m fluctuations showed the presence of four types of Ca^2+^ profiles in non-induced and LPS-induced MEFs: slowly developing decrease (SD) and increase (SI), steady-state (SS) level, and oscillations (Osc) (Fig. 7D, Panel I). Since the number of cells with Osc pattern were low (5-7%), we focused the analysis on three major types of Ca^2+^ response. Steady-state Fus1 KO cells demonstrated a slightly elevated SD-type of [Ca^2+^]m pattern (Fig. 7D, Panel II). However, in response to LPS stimulation, WT MEFs had significantly higher proportion of cells with a SD-type of response (Fig. 7D, Panel II) while Fus1 KO cells had significantly higher proportion of cells with SI-type of [Ca^2+^]m changes (Fig. 7D, Panel II).

In our previous work we suggested, based on the experimental data, that Fus1 may promote its calcium-modulating activities via mitochondrial Na^+^/Ca^2+^ exchanger (mNCX) that exports Ca^2+^ from mitochondria in exchange for Na^+^ [31]. In order to find out if an augmented [Ca^2+^]m response to LPS in Fus1 KO MEFs was due to elevated activity of mNCX, we pre-treated MEFs with CGP37157, a chemical inhibitor of mNCX. Indeed, pharmacological suppression of mNCX in LPS-treated Fus1 KO MEFs resulted in suppression of SI-type of [Ca^2+^]m changes shifting the LPS-induced response towards SS levels of [Ca^2+^]m (Fig 7D, Panel III) while in WT cells it was accompanied by a substantial rise in the proportion of cells with the SI-type response after LPS treatment (Fig. 7D, Panel III). Thus, loss of Fus1 in MEFs leads to profound LPS-induced alterations in [Ca^2+^]c and [Ca^2+^]m mediated via mNCX.

### *In silico* analysis of Fus1-coexpressing genes and Fus1-associated pathways point to their involvement in multiple aging-associated diseases

**Figure 8:**
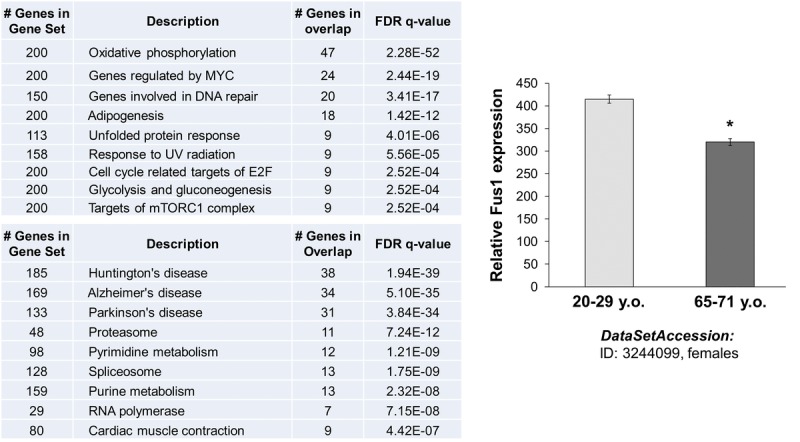
*In silico* analysis of Fus1 expression and co-expression data linked Fus1 with aging-associated diseases (**A**) Fus1 co-expression analysis revealed tight link of Fus1 with oxidative phosphorylation and with multiple neurodegenerative diseases. (**B**) Fus1 expression level is downregulated in aged female muscle tissues. *p-value ≤ 0.05 (Student's *t*-test, 2-sided unpaired). Data expressed as mean ± SEM.

We used MEM (Multi Experiment Matrix) software (http://biit.cs.ut.ee/mem/) to identify a set of human genes that strongly correlate with the Fus1 gene expression. The first 500 probes that overall recognize 386 genes showed Fus1 co-expression coefficient with p-values between 2.6e-61 and 3.2e-29. Thus, we analyzed this 386 gene set with GSEA (gene set expression analysis) software (http://software.broadinstitute.org/gsea/msigdb/annotate.jsp) to identify Fus1-associated molecular processes and diseases. Inquiry to identify the “hallmark gene sets” came up with the “oxidative phosphorylation” process as number one on the list with the *p* ≥ 4.57e-54 unequivocally confirming the association of Fus1 expression with the main mitochondrial functions - respiration and energy production. Next on the list were “DNA repair”, “adipogenesis”, “unfolded protein response”, “UV radiation response” as well as a groups of proteins involved in “glycolysis and gluco-neogenesis” (Fig. 8A). Alterations in these molecular processes are known to be closely involved in aging [33, 58-62] suggesting that Fus1 activities may be crucial in protection from premature aging. Association of Fus1 with aging was further confirmed by KEGG analysis, which identifies Huntington's, Alzheimer's, and Parkinson's diseases (Fig. 8A), the hallmark diseases of aging, as strongly correlated with Fus1-co-expression signature.

### *In silico* analysis of Fus1 expression in human muscle tissues

Furthermore, we correlated Fus1 expression in human tissues with aging. We analyzed the NCBI GEO gene expression database (www.ncbi.nlm.nih.gov/ geoprofiles) and found that Fus1 levels decline in aging muscle tissue. These data support the clinical significance of Fus1 and its link with aging that may be related to a compromised mitochondrial activity (Fig. 8B).

## DISCUSSION

Our data accumulated over several years of observations and analyses presented in this manuscript unequivocally establish the link between activities of the mitochondrial protein Fus1 and premature aging and aging-related pathologies (summarized in Table 1). Besides the data presented in this manuscript that we discuss below, we recently showed that Fus1 KO mice prematurely and progressively lose their hearing [63]. Early deterioration of hearing parameters measured in young adult Fus1 KO mice via Auditory Brainstem Response (ABR) and molecular perturbations in their cochleae were similar to the aging-related processes that occur in people of 70 years of age and older. In another recent study, we showed that adult Fus1 KO females have an early impairment in olfactory memory and spatial learning and memory [29]. These data suggested that cognitive deficiency characteristic for aging dementia patients develop in Fus1 KO mice early in life.

**Table 1:** A list of premature aging traits observed in Fus1 KO mice at the systemic, cellular and molecular levels

Systemic Features of Premature Aging
Increased mortality Low rate of fat accumulation Lordokyphosis Absence of vigory Early decline in hair re-growth Reduced ability to tolerate stress Low sperm count and reduced sperm motility Premature enlargement of seminal vesicles Altered capability of Fus1 KO adult stem cells to repopulate tissues Signs of chronic inflammation
Altered Molecular Processes Associated with Aging
Increased basal and stress-induced ROS production Intrinsic deficiency in mitochondrial respiratory reserve Perturbation in steady-state and stimuli-induced calcium homeostasis

Aging is a complex process that is influenced by both genetic and environmental factors. Premature aging phenotypes have been described in mice with targeted disruption of genes involved in control of mutation rate, regulation of chromosome and telomere stability, ROS homeostasis, apoptosis, stress responses, circadian clock, insulin/IGF and mTOR/nutrient sensing pathways, stem cells maintenance, *etc*. [35, 64-73].

The essential question is what are the critical mechanisms underlying Fus1 loss-associated early aging and age-related pathologies. We show here and in our previous studies [25, 26, 31, 32, 44] that Fus1 regulates mitochondrial health, in particular, ROS and Ca^2+^ homeostasis, and thus their dysregulation is likely to be a major mechanism that mediates Fus1-dependent aging-related processes. ROS and Ca^2+^ are two key second messengers that play critical roles in signaling cascades initiated in response to various stresses, pathogenic, nutrient and environmental signals; their dysregulation is linked to many aging-related pathologies [74-77]. Increasing evidence suggest a mutual interplay between calcium and ROS signaling systems, which seems to have important implications for fine tuning cellular signaling networks. Therefore, dysfunction in either system might affect the other system, thus potentiating harmful effects that might contribute to the pathogenesis of various disorders [78]. Therefore, Fus1 KO mice represent a unique model for studying the roles of Ca^2+^, ROS, and their interplay in systemic aging and age-associated pathologies.

We also found that other mitochondrial activities linked to ROS and Ca^2+^ homeostasis, such as respiration (oxidative phosphorylation) (Fig. 6) or AO activities [25, 31, 32] are also perturbed in Fus1 KO mice. Here, we showed that Fus1 KO cells are distinguished with low respiratory reserve capacity, RRC, a term that is used to describe the amount of extra ATP that can be potentially produced by oxidative phosphorylation in case of a sudden increase in energy demand. Depletion of the RRC has been related to a range of pathologies affecting high energy demanding tissues such as the brain, muscle, and reproductive tissues [79-81]. Age-related decrease in RRC is implicated in a variety of aging-associated pathologies [79, 82]. If the RRC is not sufficient to fuel cells with the required amount of ATP, cells risk being driven into senescence or cell death. The Bioenergetic Health Index (BHI) is a new concept in mitochondrial translational research [83]. Young and healthy subjects have a high BHI with a high RRC, high ATP-linked respiration (ALR) and low PL. Chronic metabolic stress induces damage in the mitochondrial respiratory machinery by progressively decreasing mitochondrial function and this manifests as low ALR, low RRC and high non-mitochondrial (e.g. ROS generation via NADPH oxidase and other non-mitochondrial enzymes) respiration (NMR). The persistence of dysfunctional mitochondria damages the mtDNA, which impairs the integrity of the mitochondrial biogenesis program, leading to a progressive deterioration in bioenergetic function and decreasing BHI. Our data suggest that early accumulation of bioenergetically inefficient mito-chondria resulting in increased ROS production/oxidative stress and low respiratory reserve are intrinsic properties of Fus1-deficient cells and tissues. These events may result in fast deterioration of BHI, aging, development of age-associated pathologies and premature death in Fus1 KO mice.

One of the well-documented features of aging is the increase in systemic inflammatory state, a process called inflammaging [84]. Therefore, we tested if adult Fus1 KO mice have signs of sterile chronic inflammation as was observed in our earlier study analyzing peritoneal macrophages in young mice [25]. Here, we compared a proportion of different immune cell subsets from the peripheral blood and spleen tissues of 6 m.o. WT and Fus1 KO mice. The observed significant increase in Gr1^+^ myeloid cells in the peripheral blood of Fus1 KO mice (Fig. 5) may indicate alterations in the proportion of hematopoietic precursors or their survival. Thus, SIRT1 KO mice with aging-like phenotype demonstrated a shift towards myeloid hematopoiesis [85]. Analysis of Fus1 KO spleen cell subsets also showed a significant increase in the number of Gr1+ CD11b+ F4/80 cells. This subset corresponds to inflammatory monocytes [86] and is suggestive of underlying systemic inflammation. Elevated inflam-matory monocytes expand in acute as well as chronic inflammatory conditions and in aged mice and humans [87-89]. Thus, our analysis suggests that adult Fus1 KO mice have signs of chronic inflammation that may lead to premature aging later in life.

Another aging-associated pathological attribute of Fus1 deficient tissues is an altered capability of adult stem cells to repopulate tissues, a feature that is commonly linked to early cellular and organismal senescence [90]. We inferred this based on all our previously published [26] and new data (Figs. 3 and 6), which demonstrated that stem cells compartments, such as GI crypt epithelial cells and melanocyte stem cells [26], stem cells of hair follicles (Fig. 3), and bone marrow-derived T cell progenitors (Fig. 6) have a reduced capability for tissue repopulation, suggesting that it could be one of the mechanisms of early aging of Fus1 KO mice.

Currently, it is well established that aging and age-related diseases are tightly associated with profound alterations in Ca^2+^ homeostasis [49-56, 91-94]. For example, aging β-cells showed a decrease in the amplitude of cytosolic Ca^2+^ accumulation after treatment with glucose and Ca^2+^ [56, 95]. Furthermore, β-cells from naturally and prematurely old mice demonstrated a significant reduction in the amplitude of slow and fast [Ca^2+^]c oscillations [95]. Interestingly, in Fus1 KO iKEC, Ca^2+^ dynamics was similar to Ca^2+^ dynamics in aging β-cells: ionomycin-induced Ca^2+^ accumulation occurred with a much lower amplitude in Fus1 KO than in WT cells while the time necessary to reach maximal value of response and the plateau phase were significantly shortened pointing out the deficiency in the dynamic development of Ca^2+^ response (Fig. 7). Cellular Ca^2+^ response largely depends on mitochondrial Ca^2+^ transport. By taking up Ca^2+^ from the cytosol through the mtCU (mitochondrial calcium uptake) mechanism, mitochondria maintain generation of Ca^2+^ oscillations via emptying an ER Ca^2+^ store that maintains Ca^2+^ elevations and prevents autoinhibition of Ca^2+^ channels [96-99]. Furthermore, mNCX (mito-chondrial sodium-calcium exchanger) exports Ca^2+^ from the mitochondrial matrix into the ER/mitochondria connection cleft leading to a stimulation of Ca^2+^ release from RyR (ryanodine receptors) and IP3R (inositol trisphosphate receptor), thus providing Ca^2+^ response with a positive feedback loop [100-101]. Finally, mitochondrial ROS help to coordinate processes of Ca^2+^ entry through Ca^2+^ channels and ATP-dependent Ca^2+^ transport into ER store [102].

**Figure 9:**
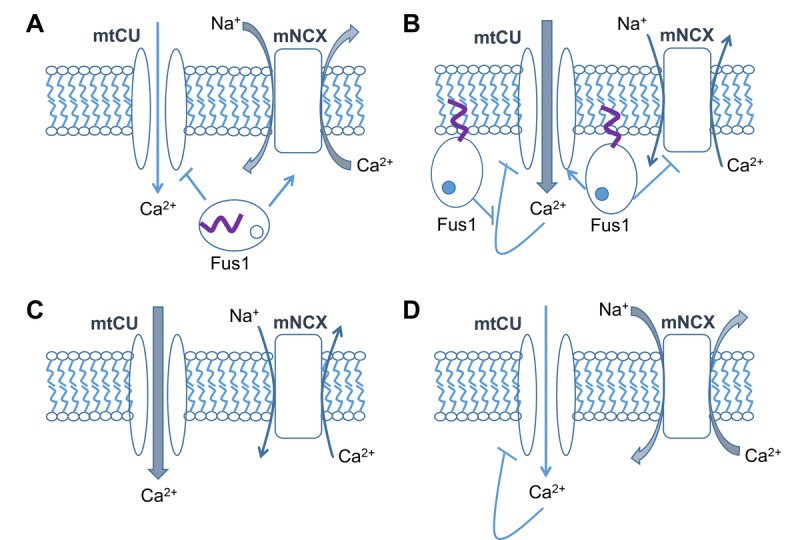
Hypothetical model of Fus1 activities in mitochondria (**A**) At steady-state or low [Ca^2+^]c levels, Fus1 has a dual effect on [Ca^2+^]m: it (1) stimulates mitochondrial Na^+^/Ca^2+^ exchanger (mNCX) that is responsible for efflux of Ca^2+^ from the mitochondrial matrix in exchange for Na^+^ from the intermembrane space, and (2) inhibits mitochondrial Ca^2+^ uptake (mtCU) mechanisms (e.g., MCU). These data allow us to consider Fus1 as a gatekeeper for mtCU, which is potentially able to filter out Ca^2+^ signals with inappropriate characteristics (e.g., low-amplitude, short, etc.). (**B**) Binding of Ca^2+^ (dark circle inside Fus1) to Fus1 after [Ca^2+^]c elevation leads to a release of myristoil residue (purple tail) and its anchoring to the mitochondrial matrix membrane. It is accompanied by mNCX inhibition and mtCU activation. The latter has an ability of self-inhibition by Ca^2+^ (negative feedback loop), the mechanism that is probably suppressed by Fus1 thereby letting mtCU to gain inward Ca^2+^ currents in a dynamic mode demonstrating a feature of a positive feedback loop. (**C**) In the absence of Fus1, mitochondria accumulate more Ca^2+^ at steady state or at the beginning of a Ca^2+^ response due to the lack of the gatekeeping function of Fus1 and decreased activity of mNCX. (**D**) During the dynamic development of a Ca^2+^ response, mtCU in mitochondria lacking Fus1 is auto-inhibited by Ca^2+^ while mNCX is activated due to the lack of Fus1 suppressive activities, which results in an elevated efflux of Ca^2+^ from mitochondria.

Earlier, we demonstrated that Fus1 protein is a potential Ca^2+^-binding protein involved in the regulation of mitochondrial Ca^2+^ transport. Based on our data [31, 44], the mechanism of Fus1's regulatory effect, most likely, includes both stimulation of mtCU and inhibition of mNCX after rising [Ca^2+^]c levels. In the absence of Fus1, mitochondrial efficiency in handling Ca^2+^ levels is impaired, thus affecting overall Ca^2+^ signaling [31]. Here, we show that in Fus1 KO ionomycin-treated epithelial cells [Ca^2+^]m levels are increased to a higher amplitude than in WT cells, but drops to a steady-state level with a faster rate. Thus, we propose that Fus1 plays a gatekeeper role for mtCU by restraining the initial accumulation of Ca^2+^ in mitochondria during Ca^2+^ load and preventing mitochondria from Ca^2+^ uptake at low cytosolic Ca^2+^ concentration (Fig. 7). In this context, it is also important to note that MICU1, another mitochondrial Ca^2+^-binding protein and Ca^2+^ sensor for MCU (Mitochondrial Calcium Uniporter), prevents autoinhibition of MCU by Ca^2+^ thereby providing mitochondrial Ca^2+^ uptake with a positive feedback loop [103]. We propose that Fus1 may play a similar function in order to maximize mito-chondrial Ca^2+^ uptake during the time course of Ca^2+^ response by suppressing Ca^2+^-induced inhibition of mtCU (Fig. 9). Positive feedback provided by Fus1 may potentially regulate the dynamics and amplitude of the [Ca^2+^]c response. The development of Ca^2+^ response in non-excitable cells such as iKEC relies on the Store-Operated Calcium Entry (SOCE) mechanism [104]. It has been recently revealed that mtCU maintains SOCE via emptying the ER Ca^2+^ store leading to oligomeric clustering of STIM1 protein, which binds in the cellular membrane to Ca^2+^ Release-Activated Ca^2+^ (CRAC) channels responsible for the SOCE [96]. Simultaneously, increased activation of mNCX in Fus1 KO cells would result in decreasing of [Ca^2+^]c accumulation while mito-chondria experience deficiency in Ca^2+^ uptake (Fig. 7).

To study the role of Fus1 in Ca^2+^ dynamics during inflammation, a critical factor in aging, we used LPS to stimulate MEFs, which robustly respond to this stimulus by transitory Ca^2+^ mobilization [57] followed by Ca^2+^ release from the ER that is required for production of cytokines [105, 106]. We demonstrated that Fus1 loss results in a shift of [Ca^2+^]c responses from oscillatory to sustained plateau-like elevation patterns, which points out to the involvement of a positive feedback loop into Ca^2+^ response regulatory circuit in Fus1 KO MEFs that was apparently suppressed in WT MEFs. Thus, we suggest that activation of Fus1 may serve as a bifurcation point between two states of Ca^2+^ response, oscillations and sustained increase.

Ca^2+^ signaling is one of the most important transducing processes and is involved in a wide range of cellular and systemic processes. It is now more than three decades since the first proposal of a “Ca^2+^ hypothesis of aging” [107]. In its mature formulation, the hypothesis provides mechanistic explanation not only for the aging process but also for Alzheimer's disease (AD) and other neurodegenerative diseases [93, 108]. Here we showed that Fus1, a small EF-hand containing mitochondrial protein, regulates multiple aspects of cellular Ca^2+^ homeostasis and is involved in aging and age-associated pathologies. These data, combined with our recent data on early hearing loss and impairment in olfactory and spatial memory in Fus1 KO mice [29, 30], suggest that the mechanism of Fus1 loss-mediated premature aging involves impaired homeostasis of [Ca^2+^]c and [Ca^2+^]m as well as dysregulation in ROS and ATP production. Since mitochondrial permeability transition pore (mPTP) opening depends on ROS and mitochondrial Ca^2+^ elevations [109, 110], we suggest that one of the mechanisms of Fus1 protection against premature aging is regulation of mPTP opening. Indeed, ROS-induced oxidation of mPTP components such as adenine nucleotide translocator (ANT) and Cyclophilin D (CypD) along with the increased Ca^2+^ levels in mitochondrial matrix promote channel opening [111]. mPTP opening leads to mitochondrial membrane potential dissipation, loss of small molecules (for example, reduced glutathione), and release of ROS and Ca^2+^ from mitochondria [109] that may lead to apoptosis, blocking of cell repopulation and tissue regeneration. Indeed, we showed that *Fus1* KO mice are prone to early development of neurodegenerative diseases such as hearing loss and dementia. [29, 30]. Earlier, we demonstrated that in Fus1 KO CD4^+^ T cells mPTP state was more opened at 4 hours post- activation as compared to WT CD4^+^ T cells [31]. Strategies targeting mPTP in neurodegenerative diseases such as AD or Parkinson's disease have been recently designed for further clinical evaluation [112].

Finally, it has been reported that fine-tuning of mtCU by MICU1, a mitochondrial Ca^2+^-binding protein that regulates MCU and has multiple similarities to the Fus1 protein, leads to proliferation of hepatocytes in response to regeneration signals after partial hepatic-tomy. On the other hand, lack of MICU1 induces mitochondrial Ca^2+^ overload triggering cell death and tissue injury [113]. Our findings that Fus1 KO mice demonstrate signs of premature aging such as a decreased hair re-growth ability and lack of adequate amount of stem cells to repopulate tissues go in line with connection of mtCU and tissue regeneration established for MICU1.

Future studies will clarify the detailed roles of Fus1 and other mitochondrial components that regulate Ca^2+^ response in the natural aging process in mice and humans. Improved understanding of the interconnection between aging and mitochondrial Ca^2+^ and ROS homeostasis, identification of Fus1 transcriptional targets specifically involved in aging, and new drugs that modulate particular Fus1-dependent aspects of Ca^2+^ signaling may ultimately lead to the development of novel strategies to prevent and/or treat age-related pathologies.

## METHODS

### Ethics statement

Investigation has been conducted in accordance with the ethical standards and according to the Declaration of Helsinki and according to national and international guidelines and has been approved by the authors' institutional review board

### Fus1 KO mice

Fus1 KO mice generated by Dr. A Ivanova [23] were backcrossed to 129sv background in the laboratory of Dr. S Anderson (NCI-Frederick). All animal experi-ments were performed according to a protocol approved by the Yale University Institutional Animal Care and Use Committee (IACUC) and the animals were cared for according to the recommendations in the “Guide for the Care and Use of Laboratory Animals” (National Institutes of Health). The mice were fed with a standard diet, housed in standard cages, (five per cage) and maintained on a 12 h light–dark cycle. They had *ad libitum* access to drinking water and normal diet throughout the experiment.

### BrdU proliferation analysis

Mice were injected *i.p*. with 100 μg of 5-bromo-2-deoxyuridine once every day for 2 days. On the third day, mice received two injections 4 hours apart. 24 hours later, thymocytes were isolated, counted using trypan blue, and stained for CD4 and CD8 surface molecules using fluorochrome-coupled antibodies. Cells were then stained with anti-BrdU antibody following conventional methodology. Cells were analyzed in FACS Calibur instrument.

### Preparation and characterization of WT and Fus1 KO spermatozoa

Male mice were euthanized by CO_2_ asphyxiation. For preparation of spermatozoa, caudae epididymis and vasa deferentia were excised, rinsed with buffer and transferred to 1 ml Tyrode's medium. Semen was allowed to exclude from 3 to 5 small incisions (10 min at 37°C, 5% CO_2_). Sperm count and motility of the sperm cells were analyzed with CEROS computer-assisted semen analysis system (version 10; Hamilton Thorne Inc., Beverly, MA, USA). For assays, sperm cells were kept in the indicated media for no longer than 15 min.

### Measurement of mitochondrial respiration

We used high resolution respirometry oxygraph-2k (Oroboros Instruments) to measure mitochondrial respiration. Two types of cells were used in the study: WT and Fus1 KO primary MEFs of 2^nd^ passage and spontaneously immortalized kidney epithelial cells (iKEC). Equal number of cells were plated 24 hours before OCR measurement. For each run 4 million cells/ml were used. After measurement of basal OCR, different drugs inhibiting mitochondrial respiration were added sequentially. The following drugs were used: oligomycin, a complex V (ATPsyntase) inhibitor (1 μg/ml) that significantly reduces electron flow through the electron transport chain (ETC); FCCP, (carbonyl cyanide-p-trifluoromethoxyphenyl-hydrazon), a protonophore that collapses the inner membrane proton gradient allowing the ETC to function at its maximal rate (1 μM); antimycin A and rotenone (10 μM) (inhibitors of complex III and I, respectively) were added to shut down ETC function, revealing the non-mitochondrial respiration. The basal respiration (BR) rate was determined by the difference between the starting OCR (Oxygen Consumption Rate) and NMR (Non-Mitochondrial Respiration). NMR is OCR after shutting down ETC function with antimycin A and rotenone. The proton leak (PL) rate was determined by the difference between the OCR after adding oligomycin and the OCR after adding antimycin/rotenone mixture (NMR). The ATP linked respiration was determined by the difference between the starting OCR and the OCR after adding oligomycin. Maximal respiration (MR) is derived by subtracting NMR from the OCR after adding FCCP. Mitochondrial reserve capacity is calculated by subtracting basal respiration from maximal respiratory capacity. Experiments were repeated at least 3 times.

### Flow cytometry of immune cell subsets from peripheral blood and spleen

Lymphocytes and myeloid cells were analyzed by multi-color flow cytometry using LSRII flow cytometer and FACS Diva software to analyze the obtained data. To estimate the content of macrophage fraction in the spleen, we used CD11b and F4/80 antibodies (BioLegend). For further characterization of this lineage, we used antibodies to Ly6C and Gr1 (eBioscience) to distinguish surface profiles of potential myeloid-derived suppressor cells, monocytes, and macrophages. Also, we used specific markers for T (CD3, CD4, CD8) and B (B220) cells, NK lymphocytes (DX5), myeloid (Gr1) and dendritic (CD11c) cells to additionally characterize immune cell content (antibodies were obtained from eBioscience or BD PharMingen). Splenocytes and blood cells were stained with antibodies for 20 min at 4°C, washed out twice and used for analysis as described elsewhere.

### Ca^2+^ dynamics studies

To compare the Ca^2+^ responses of WT and Fus1 KO iKEC or mouse embryonic fibroblasts (MEFs) to Ca^2+^-inducing agents, we used the Ca^2+^ agonist Ionomycin (1 μM) (Sigma) and mouse recombinant LPS (100ng/mL) for epithelial cells and fibroblasts, respectively. For studying changes in mitochondrial calcium ([Ca2+]m), fresh epithelial cells were grown overnight on 8-chamber coverglass slides (Lab-Tek, Nalge Nunc International). The following day, cells were simultaneously treated *in situ* with the AM ester of Ca^2+^ sensor dye rhod-2 (5 μM; Kd = 570 nM) (AnaSpec, Fremont, CA) and mitochondria-specific fluorescent marker MitoTracker Green (MTG; 150 nM) (Molecular Probes) at 37°C for 30 min ([114, 115]), washed out with phosphate-buffered saline (PBS) and mounted. Rhod2/MTG co-localization was studied by scanning at 488 and 543 nm while monitoring fluorescence for MTG and Rhod-2 at 515 and 600 nm, respectively. Image acquisition was made with the software NIS Elements Advanced Research for Nikon TE2000-U C1 confocal laser scanning microscope (Nikon Instruments Inc., Melville, NY). Obtained values of Rhod-2 fluorescence were normalized to the level of MTG fluorescence to avoid false positive or negative results associated with movement of mitochondria relative to the focus of the microscope. Time series data for [Ca2+]m were presented as a ratio of normalized Rhod-2 fluorescence (F) to the basal level of Rhod-2 signal (Fo).

For measurement of [Ca2+]c, we utilized ratiometric Ca^2+^ sensor Fura-2. Cells were grown in a similar manner as described for [Ca2+]m analysis. The next day, cells were treated with 1 μM fura 2-AM (Molecular Probes) for 30 min at 37°C [116].]. Ca^2+^ imaging was performed using a Nikon TE2000-E wide-field fluorescence microscope equipped with a xenon lamp for fluorescence excitation. Fura 2-loaded cells were excited using 340 and 380 excitation filters and a polychroic mirror. Fluorescence was captured by a CoolSNAP HQ2 camera (Roper Scientific). Ratio measurements (340/380) were recorded at 5-s intervals over a 20-min time period. The [Ca2+]c concentration for cells treated with Ca^2+^ agonists was estimated by 340/380 ratio (F) normalized to the basal level of corresponding 340/380 ratio in the same cells (Fo).

## SUPPLEMENTARY MATERIALS VIDEO





## Abbreviations

ALR: ATP-linked respiration
AO: antioxidant
BR: basal respiration
Ca^2+^: calcium ions
[Ca^2+^]c: cytoplasmic Ca^2+^
[Ca2+]m: mitochondrial Ca^2+^
ETC: electron transport chain
iKEC: immortalized kidney epithelial cells
LPS: lipopolysaccharide
MDSC: myeloid-derived suppressor cells
MEF: mouse embryonic fibroblasts
mNCX: mitochondrial sodium-calcium exchanger
MCU: mitochondrial calcium uniporter
m.o.: months old
mPTP: mitochondrial permeability transition pore
mtCU: mitochondrial calcium uptake
MR: maximal respiration
MTG: MitoTracker Green
NMR: non-mitochondrial respiration
PL: proton leak
ROS: reactive oxygen species
RRC: reserve respiratory capacity
SOCE: store-operated calcium entry.
