# Supplementary material for: Mitochondrial protein Fus1/Tusc2 in premature aging and age-related pathologies: critical roles of calcium and energy homeostasis
Source: Aging (Albany NY). 2017 Mar 26;9(3):627–48. doi: 10.18632/aging.101213 (PMC5391223; doi:10.18632/aging.101213)
Supplement: Supplementary file 1 [file aging-09-627-s001.pdf]

SUPPLEMENTARY MATERIAL

Please browse the Full text version to see Supplementary Video "Multiple signs of premature aging in Fus1 KO mice". Video file showing 15 mo old Fus1 KO mouse with characteristic for this age absence of vigor, hunchback, fat and muscle wasting. Shaved area that was not closed at 3 months after shaving is also visible.
